# Associations of urinary phthalate metabolites with Circadian Syndrome: evidence from NHANES

**DOI:** 10.3389/fpubh.2025.1597489

**Published:** 2025-06-27

**Authors:** Chunxing Yi, Jie Shen, Jiansheng Cai

**Affiliations:** ^1^Laboratory Center, Guangxi Health Commission Key Laboratory of Glucose and Lipid Metabolism Disorders, The Second Affiliated Hospital of Guilin Medical University, Guilin, China; ^2^Department of Preventive Health Care, Liuzhou Traditional Chinese Medical Hospital, Liuzhou, China; ^3^Department of Environmental Health and Occupational Medicine, School of Public Health, Guilin Medical University, Guilin, China

**Keywords:** phthalates, Circadian Syndrome, BKMR, quantile g-computation, NHANES

## Abstract

**Background:**

The relationship between phthalate exposure and circadian rhythm disruption lacks epidemiological evidence. This study investigated the association between exposure to ten phthalates (PAEs) and Circadian Syndrome (CircS) among American adults.

**Methods:**

Data from the 2013–2018 United States National Health and Nutritional Health Surveys (*N* = 2519) were analyzed using logistic regression to assess associations between individual phthalate exposure and CircS. Restricted cubic splines (RCS) evaluated dose-response relationships, while Bayesian kernel machine regression (BKMR) and g-computation models assessed the effects of phthalate mixtures.

**Results:**

The prevalence of CircS in the study population was 45.14%. Participants in the fourth quartile of exposure to MECP phthalate (OR = 1.632, 95% CI: 1.159–2.300), MEHP phthalate (OR = 1.830, 95% CI: 1.301–2.573), mono-benzyl phthalate (OR = 1.699, 95% CI: 1.156–2.496), and MEOH phthalate (OR = 1.560, 95% CI: 1.065–2.279) had an increased risk of CircS compared to those in the first quartile of exposure. RCS analysis indicated a linear positive association between exposure to MECP, MEHP, and mono-benzyl phthalate and CircS risk. BKMR and quantile g-computation analyses demonstrated that combined phthalate exposure was positively associated with CircS.

**Conclusion:**

Individual and mixed exposures to certain phthalates may increase the risk of CircS, providing evidence for prevention strategies targeting endocrine-disrupting chemicals.

## Introduction

The 24-h daily cycle in humans governs physiological functions like energy conversion and signal transmission by detecting external alterations. If the circadian timing is out of sync and disturbed, it could result in diverse metabolic and physiological disruptions (1). The disturbance of circadian rhythms is not just a significant potential contributor to the elements of metabolic syndrome, such as abdominal obesity, raised blood pressure, higher triglyceride levels, reduced high-density lipoprotein cholesterol, and hyperglycemia (2–5), but is also associated with the main comorbidities of metabolic syndrome, such as sleep disorders and depression. Based on this, researchers believe that circadian rhythm disruption may be a significant potential causative factor of metabolic syndrome and have named it “Circadian Syndrome (CircS)” (6). CircS incorporates depression and sleep disturbances into the established criteria for metabolic syndrome (MetS), signifying a novel condition of cardiovascular and metabolic dysfunction that offers a more accurate prediction of cardiovascular disease than conventional MetS (7).

Environmental endocrine disruptors (EDCs) are external chemicals that can disrupt hormone functions (8). Contact with EDCs might result in alterations of locomotor and behavioral patterns by modulating the expression levels of key clock and circadian rhythm network genes (9). Phthalates (PAEs) constitute a prevalent category of EDCs that are extensively employed as plasticizers in the manufacturing of plastic goods to augment their pliability, processability, and malleability (10). Moreover, PAEs are also broadly applied in the production of pharmaceuticals, cosmetics, and personal care items to improve the efficacy and quality of these products (11). These compounds are prone to leaching from products, thereby posing a risk of contamination in workplaces, indoor settings, and food items. People may come into contact with PAEs via multiple pathways, such as through food consumption, breathing in contaminated air, and skin contact (12).

Experimental studies have revealed the potential impacts of phthalate exposure on circadian rhythms. For example, early developmental exposure to di(2-ethylhexyl) phthalate (DEHP) disrupts the biological clock in Caenorhabditis elegans (13). Similarly, in Drosophila development, exposure to dibutyl phthalate (DBP) inhibits the expression of key genes that regulate circadian rhythms (11). Moreover, after oral exposure to DEHP, the expression patterns of circadian rhythm genes in the livers of mice are also disrupted (14). Nevertheless, existing epidemiological research has not yet yielded conclusive evidence to definitively link phthalate exposure with circadian rhythm disturbance.

Considering that PAEs can potentially disrupt circadian rhythms, this research leveraged data from the National Health and Nutrition Examination Survey (NHANES) to examine the relationship between exposure to individual phthalates and exposure to phthalate combinations with CircS. The objective of this study was to deepen the comprehension of the connection between PAEs and CircS.

## Materials and methods

### Study design and population

**Figure 1:**
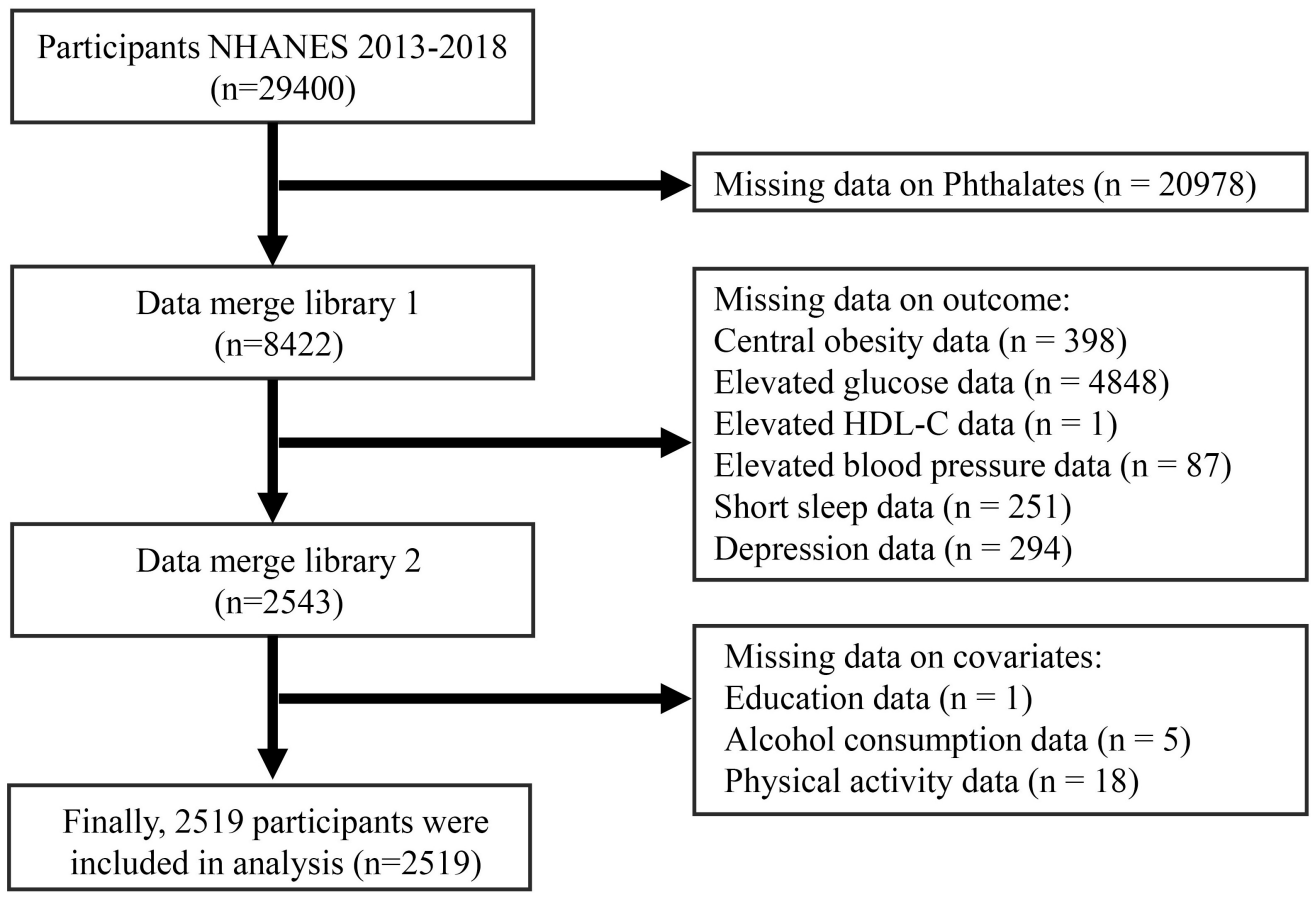
Flow chart for inclusion of study participants.

The National Health and Nutrition Examination Survey (NHANES) is a nationwide cross-sectional study administered by the National Center for Health Statistics (NCHS), which is affiliated with the Centers for Disease Control and Prevention (CDC). It utilizes a multistage random probability sampling method. The research ethics were approved by the NHANES review board of the Centers for Disease Control and Prevention. For this study, we drew upon data from three survey cycles of NHANES that took place between 2013 and 2018 to explore the link between PAEs exposure and CircS in adults. Following the screening of comprehensive examination records, laboratory results, and questionnaire information, a total of 2,519 participants were ultimately included in the analysis. Figure 1 shows the flowchart of participant inclusion and exclusion.

### Exposure assessment

In the NHANES 2013–2018 dataset, 13 phthalate metabolites were identified, comprising Mono(carboxyisononyl) phthalate, Mono(carboxyisoctyl) phthalate, Mono-2-ethyl-5-carboxypentyl phthalate (MECP phthalate), Mono-n-butyl phthalate, Mono-(3-carboxypropyl) phthalate, Mono-ethyl phthalate, Mono-(2-ethyl-5-hydroxyhexyl) phthalate (MEHP phthalate), Cyclohexane 1,2-dicarboxylic acid monohydroxy isononyl ester (MHNCH), Mono-(2-ethyl)-hexyl phthalate, Mono-isobutyl phthalate, Mono-isononyl phthalate, Mono-(2-ethyl-5-oxohexyl) phthalate (MEOH phthalate), Mono-benzyl phthalate. Comprehensive details regarding the chemical measurement methodologies are accessible through the NHANES Laboratory Data module (https://wwwn.cdc.gov/nchs/nhanes/Default.aspx). The limits of detection (LOD) varied between 0.2 and 1.2 ng/mL. For the ensuing analyses, only phthalate metabolites with detection frequencies exceeding 60% were incorporated (15), prompting the exclusion of MHNCH, Mono-(2-ethyl)-hexyl phthalate, and Mono-isononyl phthalate (with sample detection rates of 46.2%, 53.7%, and 22.9%, respectively). The detection rates of the remaining phthalate metabolites ranged from 84.2% to 99.88%. Values below the detection threshold were substituted with the lower limit of detection divided by the square root of 2.

### CircS assessment

Anthropometric and biochemical assessments were performed by qualified staff at the Mobile Examination Center (MEC) and through home visits. The CircS is characterized by the presence of four or more of the following seven components (7): (1) increased waist circumference (≥ 88 cm for females, ≥ 102 cm for males); (2) elevated fasting glucose (≥ 100 mg/dL) or on pharmacological treatment for hyperglycemia; (3) elevated serum triglycerides (≥ 150 mg/dL) or on pharmacological treatment for hypertriglyceridemia; (4) reduced high-density lipoprotein cholesterol (HDL-C < 40 mg/dL for males; HDL-C < 50 mg/dL for females) or patients being treated with medication; (5) elevated blood pressure (≥ 130 mmHg systolic or ≥ 85 mmHg diastolic) or on pharmacological treatment for hypertension; (6) short sleep duration (< 6 h per day); (7) depressive symptoms as indicated by a Patient Health Questionnaire (PHQ-9) score of ≥ 5. The PHQ-9 is a nine-item screening tool for depression, designed to assess the presence of depressive symptoms within the past 2 weeks, with scores categorized as none/mild depression (0–4 points) or moderate/severe depression (≥ 5 points) (16).

### Covariates

Data on participants' age, sex, ethnicity, educational attainment, alcohol consumption habits, cotinine levels, and physical activity were gathered through questionnaires, physical examinations, and laboratory tests. Race was classified into categories including non-Hispanic White, non-Hispanic Black, Mexican American, and other races (including other Hispanic and multiracial individuals). Educational attainment was classified into two groups: “less than high school” (including 12th-grade students without a diploma) and “high school and above”. The assessment of drinking habits was based on the question “Had at least 12 alcoholic drinks in the past year,” which included data from both current drinkers and non-drinkers. Cotinine concentrations were employed to assess the degree of tobacco exposure among individuals; in this study, cotinine levels were divided into two categories: below 0.05 ng/mL (low) and ≥ 0.05 ng/mL (high) (17). Metabolic Equivalent of Task (MET) was utilized to determine physical activity levels, with the calculation formula for physical activity given as: Physical Activity (MET-h/week) = MET value × frequency per week × duration of each activity. The classification criteria for physical activity levels are as follows: 0 MET-h/week represents no activity, below 30 MET-h/week is defined as low activity level, 30 to 60 MET-h/week is defined as moderate activity level, and 60 MET-h/week and above is defined as high activity level (18).

### Statistical analysis

Categorical variables are presented as counts (N) and proportions (%). For continuous variables that do not conform to a normal distribution, the median and interquartile range (IQR) are utilized. Comparisons between groups for categorical variables were conducted using the Pearson chi-square test, whereas differences in continuous variables were examined using the Wilcoxon rank-sum test. A logistic regression model was employed to assess the association between PAEs and CircS, taking into account the complex survey design of the NHANES data by utilizing appropriate sampling weights. PAEs were categorized into four groups based on quartiles (Q1, Q2, Q3, and Q4), with Q1 as the reference group, and the strength of the association was determined through odds ratios (OR) and confidence intervals (CI). The initial model included each PAE without any adjustments, while subsequent models adjusted for age, gender, race, education level, drinking habits, cotinine levels, and physical activity. A restricted cubic spline (RCS) analysis was conducted to evaluate the dose-response relationship between the log_10_-transformed individual PAEs and CircS, using the 25th percentile as the reference value. The associations between the mixture of 10 log_10_-transformed PAEs and CircS were analyzed by Bayesian kernel machine regression (BKMR) and quantile g-computation models. The significance threshold for this study was established at 0.05, and all analyses were conducted using R (version 4.4.0). The BKMR and quantile g-computation models were implemented using the R packages “bkmr” and “qgcomp” respectively.

## Results

### Baseline characteristics of CircS and PAEs metabolites

Table 1 presents the baseline characteristics of the study subjects. The average age of the participants was 48.85 ± 0.64 years, comprising 1,249 females and 1,270 males.

**Table 1:** Characteristics of study population (*N* = 2519), NHANES, USA, 2013–2018.

**Characteristic**	**Total (*n* = 2,519)**	**CircS (*n* = 1,137)**	**Non-CircS (*n* = 1,382)**	***P* value**
Age (year)	48.85 ± 0.64	57.09 ± 0.65	43.02 ± 0.80	< 0.001
Gender				0.402
Male	1,249 (50.87%)	531 (49.29%)	718 (51.99%)	
Female	1,270 (49.13%)	606 (50.71%)	664 (48.01%)	
Education				0.077
Below high school	564 (14.47%)	278 (16.23%)	286 (13.22%)	
High School or above	1,955 (85.53%)	859 (83.77%)	1,096 (86.78%)	
Race				0.69
Non-Hispanic White	940 (65.54%)	430 (66.45%)	510 (64.90%)	
Non-Hispanic Black	565 (11.45%)	255 (11.25%)	310 (11.59%)	
Mexican American	369 (8.39%)	180 (8.56%)	189 (8.28%)	
Other Race	645 (14.62%)	272 (13.74%)	373 (15.24%)	
Physical activity				< 0.001
Inactive	627 (20.48%)	358 (25.96%)	269 (16.59%)	
Low activity	817 (33.35%)	400 (37.96%)	417 (30.09%)	
Medium activity	355 (16.51%)	125 (12.51%)	230 (19.33%)	
High activity	720 (29.67%)	254 (23.57%)	466 (33.98%)	
Cotinine				0.031
< 0.05 ng/mL	1,377 (57.88%)	649 (60.81%)	728 (55.81%)	
≥ 0.05 ng/mL	1,142 (42.12%)	488 (39.19%)	654 (44.19%)	
Alcohol consumption				0.005
Yes	1,550 (67.83%)	646 (63.87%)	904 (70.63%)	
No	969 (32.17%)	491 (36.13%)	478 (29.37%)	
Central obesity				< 0.001
Yes	1,496 (59.31%)	955 (84.61%)	541 (41.42%)	
No	1,023 (40.69%)	182 (15.39%)	841 (58.58%)	
Elevated glucose				< 0.001
Yes	1,588 (59.59%)	1,012 (87.95%)	576 (39.53%)	
No	931 (40.41%)	125 (12.05%)	806 (60.47%)	
Elevated triglyceride				< 0.001
Yes	1,236 (47.93%)	971 (87.19%)	265 (20.16%)	
No	1,283 (52.07%)	166 (12.81%)	1,117 (79.84%)	
Low HDL-C				< 0.001
Yes	1,305 (49.26%)	998 (87.78%)	307 (22.02%)	
No	1,214 (50.74%)	139 (12.22%)	1,075 (77.98%)	
Elevated blood pressure				< 0.001
Yes	1,358 (48.50%)	948 (80.69%)	410 (25.74%)	
No	1,161 (51.50%)	189 (19.31%)	972 (74.26%)	
Short sleep				< 0.001
Yes	285 (8.71%)	188 (13.77%)	97 (5.13%)	
No	2,234 (91.29%)	949 (86.23%)	1,285 (94.87%)	
Depression				< 0.001
Yes	653 (23.81%)	427 (35.71%)	226 (15.39%)	
No	1,866 (76.19%)	710 (64.29%)	1,156 (84.61%)	

The characteristics of ten PAEs metabolites are detailed in Supplementary Table S1. The average concentrations of PAEs ranged from 1.20 to 29.90 ng/mL. Mono-ethyl phthalate exhibited the highest concentration, followed by Mono-n-butyl phthalate and MECP phthalate, with average concentrations of 29.90 ng/mL, 9.70 ng/mL, and 8.60 ng/mL, respectively. The exposure level of MECP phthalate in CircS patients was significantly higher than that in individuals without CircS (9.00 vs. 8.30 ng/mL, *P* < 0.05).

### Association between PAEs metabolites exposure and CircS

**Figure 2:**
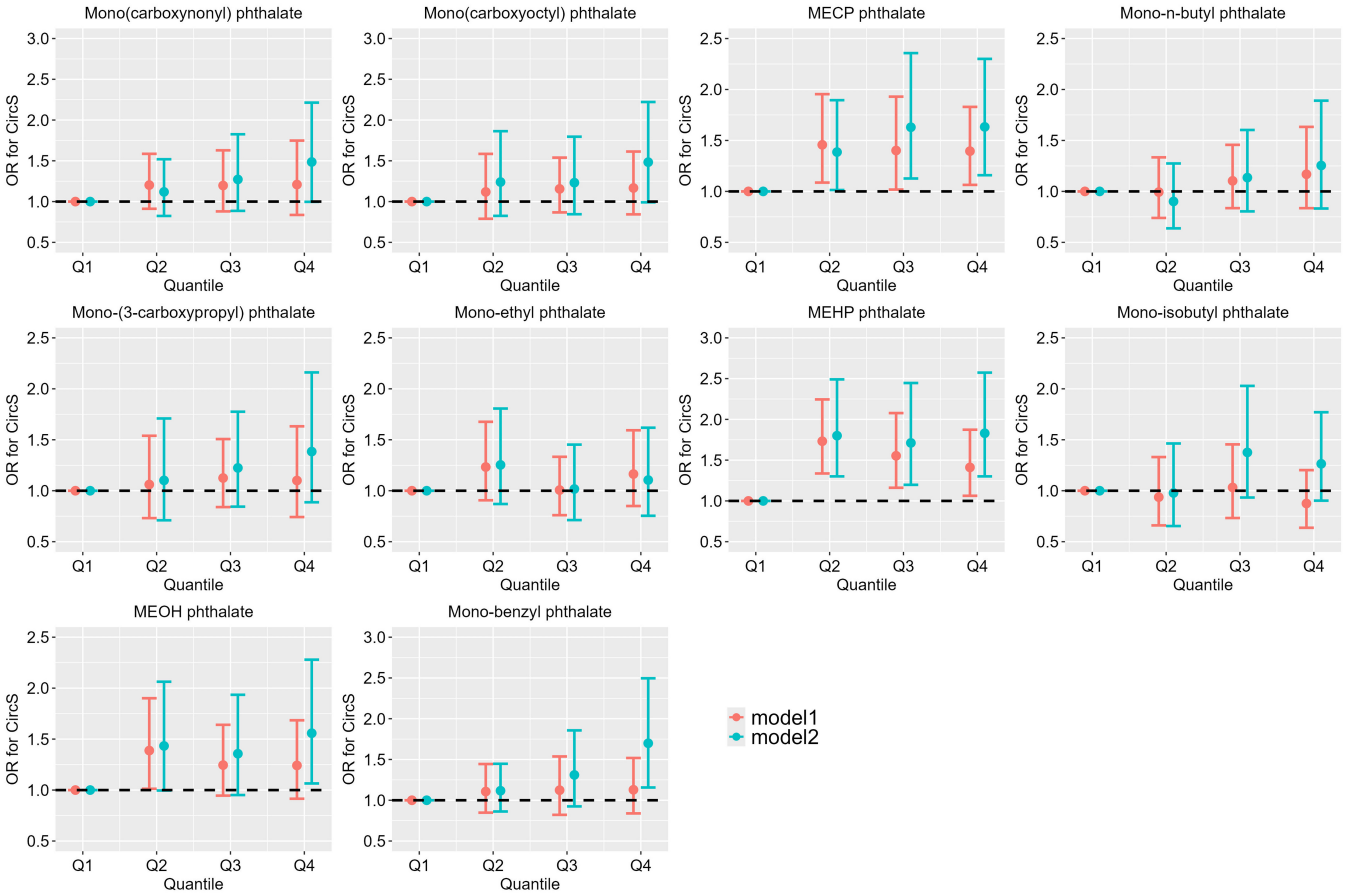
Association between single urinary phthalate metabolite concentration and CircS (*N* = 2519). NHANES, USA, 2013–2018. Model 1: The unadjusted model. Model 2: Adjusted for age, gender, educational attainment, race, alcohol consumption, physical activity, and cotinine levels.

Figure 2 summarizes the relationship between phthalate metabolites exposure and CircS. In univariate logistic regression analysis, higher exposure levels of MECP phthalate (Q2 vs. Q1: OR = 1.457, 95% CI: 1.086, 1.954; Q3 vs. Q1: OR = 1.401, 95% CI: 1.017, 1.930; Q4 vs. Q1: OR = 1.395, 95% CI: 1.064, 1.829) and MEHP phthalate (Q2 vs. Q1: OR = 1.732, 95% CI: 1.337, 2.245; Q3 vs. Q1: OR = 1.553, 95% CI: 1.162, 2.078; Q4 vs. Q1: OR = 1.411, 95% CI: 1.063, 1.873) were significantly associated with an increased risk of CircS compared to the Q1 group. After adjusting for age, gender, educational attainment, race, alcohol consumption, physical activity, and cotinine, the elevated exposure levels of MECP phthalate (Q2 vs. Q1: OR = 1.386, 95% CI: 1.013, 1.894; Q3 vs. Q1: OR = 1.630, 95% CI: 1.127, 2.357; Q4 vs. Q1: OR = 1.632, 95% CI: 1.159, 2.300) and MEHP phthalate (Q2 vs. Q1: OR = 1.800, 95% CI: 1.300, 2.490; Q3 vs. Q1: OR = 1.712, 95% CI: 1.198, 2.446; Q4 vs. Q1: OR = 1.830, 95% CI: 1.301, 2.573) remained significantly associated with an increased risk of CircS. Additionally, higher exposure levels of MEOH phthalate (Q4 vs. Q1: OR = 1.56, 95% CI: 1.065, 2.279) and Mono-benzyl phthalate (Q4 vs. Q1: OR = 1.699, 95% CI: 1.156, 2.496) also significantly increased the risk of CircS compared to the Q1 group. Supplementary Figures S1–S7 summarize the relationship between phthalate metabolites exposure and the components of CircS. Participants in the fourth quartile of exposure to MECP phthalate, MEHP phthalate, MEOH phthalate, and mono-benzyl phthalate exhibited a significantly increased risk of central obesity and elevated glucose compared with those in the first quartile of exposure (Supplementary Figures S1, S2). Additionally, participants in the fourth quartile of MECP phthalate exposure had a higher risk of low HDL-C and depression than those in the first quartile (Supplementary Figures S4, S7). Similarly, participants in the fourth quartile of MEHP phthalate exposure had a higher risk of low HDL-C than those in the first quartile (Supplementary Figure S4). Participants in the fourth quartile of mono-benzyl phthalate exposure also had an increased risk of depression compared to those in the first quartile (Supplementary Figure 7).

**Figure 3:**
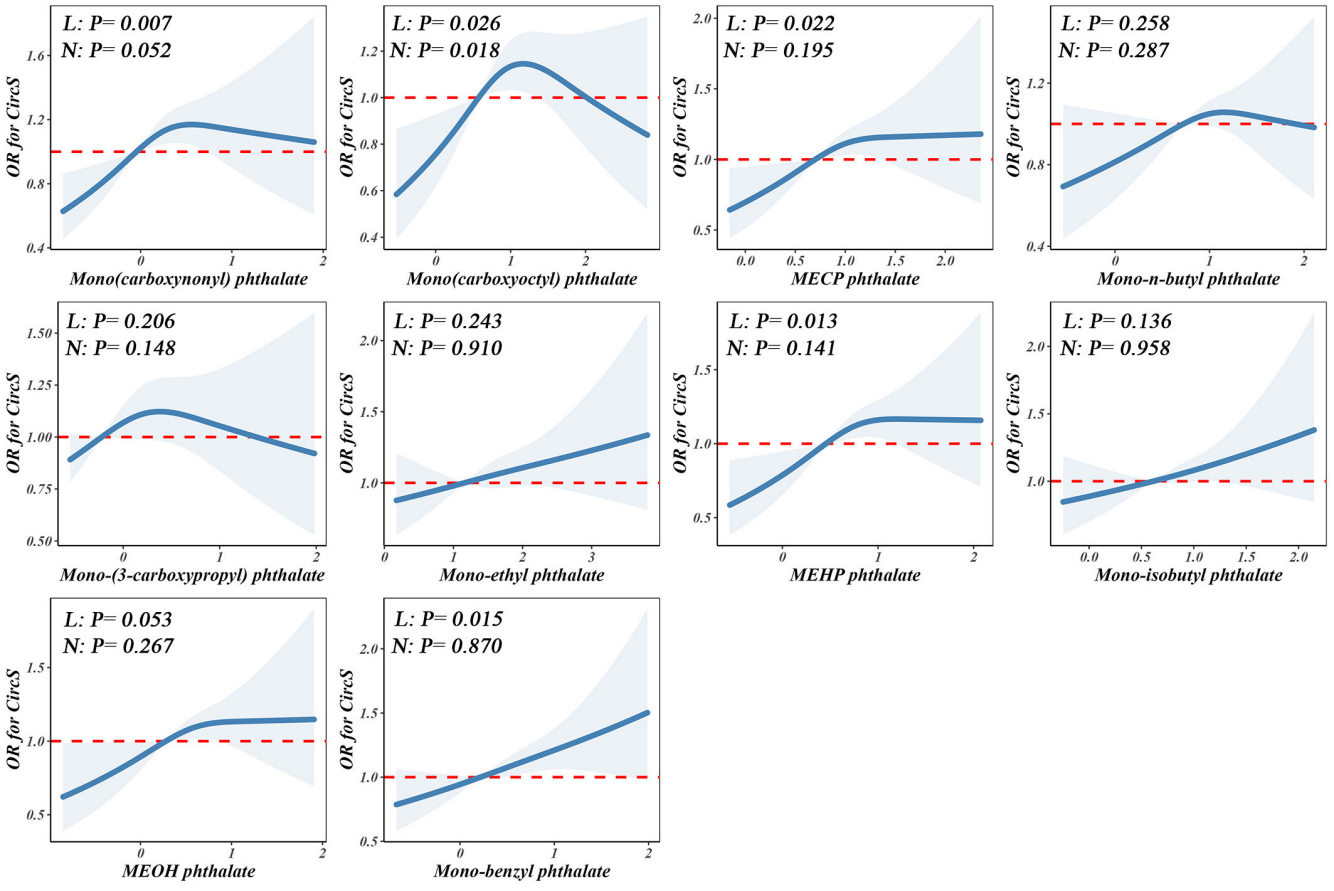
Non-linear dose–response analysis on PAEs and CircS. Restricted cubic spline regression with 3 knots at the 10th, 50th, and 90th percentiles was used to explore the potential dose–response relationship between log10-transformed PAEs and CircS, with the 25th percentile serving as the reference. Model adjusted for age, gender, educational attainment, race, alcohol consumption, physical activity, and cotinine levels. Solid lines indicate OR, and the shadow shapes indicate 95% CIs. L: P for overall; N: P for nonlinearity.

Figure 3 depicts the linear and nonlinear relationships between individual PAE exposures in urine and CircS based on RCS regression. The analysis revealed a linear positive correlation between Mono(carboxynonyl) phthalate, MECP phthalate, MEHP phthalate, and Mono-benzyl phthalate with CircS (*P*_overall_ < 0.05, *P*_nonlinearity_ > 0.05). Conversely, Mono(carboxyoctyl) phthalate exhibited a nonlinear relationship with CircS (*P*_overall_ < 0.05, *P*_nonlinearity_ < 0.05).

### Bayesian kernel machine regression analyses

**Figure 4:**
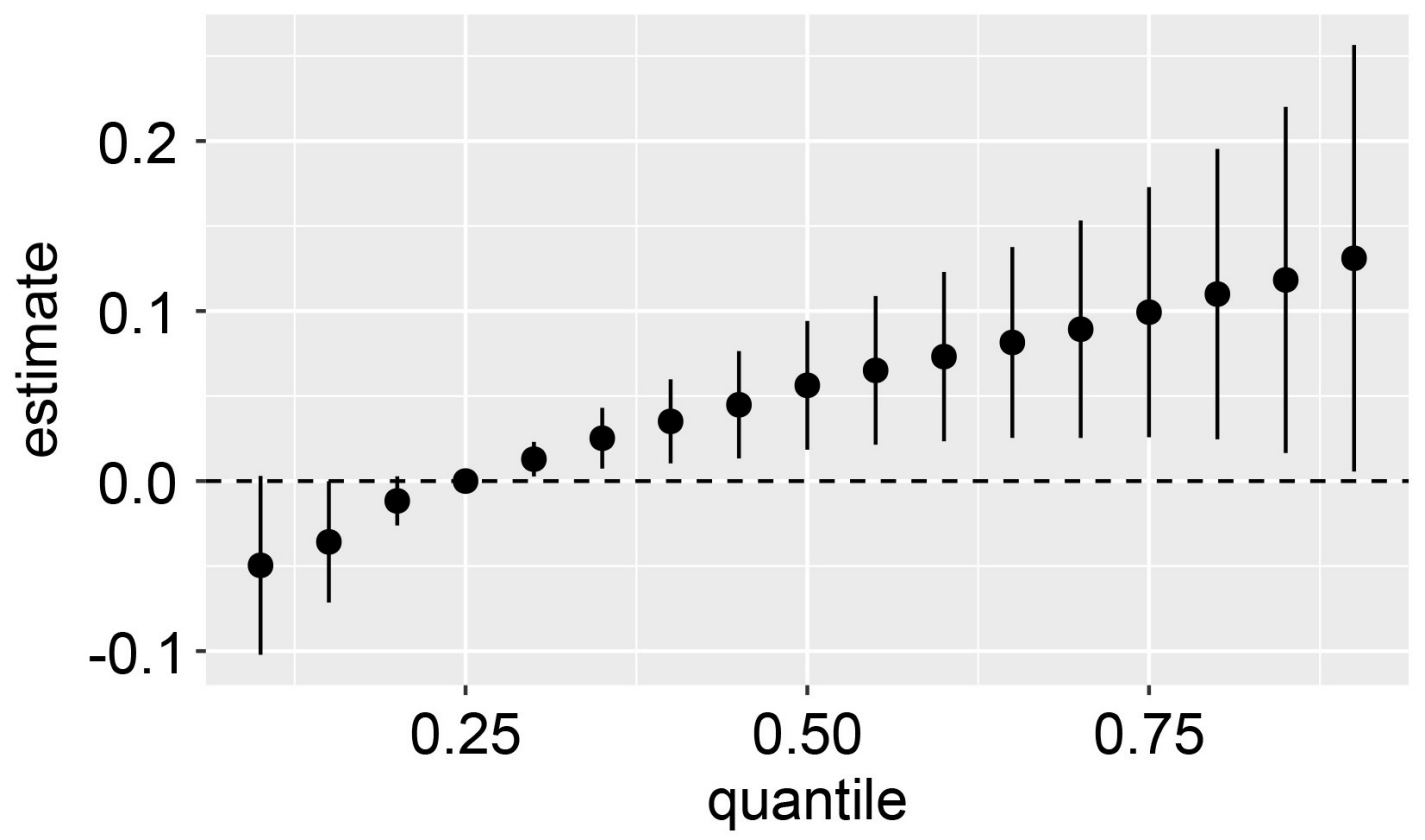
Joint effect (95% CI) of the mixture on CircS when all the PAEs at particular percentiles were compared to all the PAEs at their 25th percentile. The results were assessed by the BKMR model, adjusted for age, gender, educational attainment, race, alcohol consumption, physical activity, and cotinine levels.

Using the BKMR model, an overall association analysis of the mixtures revealed a joint association between PAE mixtures and CircS. When all metal concentrations were above the 25th percentile, the metal mixture demonstrated a significant positive correlation with CircS levels. Conversely, when all metal concentrations were below the 25th percentile, a significant negative correlation was observed compared to when all metal concentrations were at the 25th percentile (Figure 4). Moreover, the BKMR model was employed to explore the potential exposure-response relationships while maintaining other PAEs levels at their corresponding 25th percentiles. The findings from the BKMR model aligned with the results derived from the RCS model, indicating a linear positive correlation between Mono(carboxynonyl) phthalate, MECP phthalate, MEHP phthalate, and Mono-benzyl phthalate with CircS (Supplementary Figure S8).

### Quantile G-Computation model

**Figure 5:**
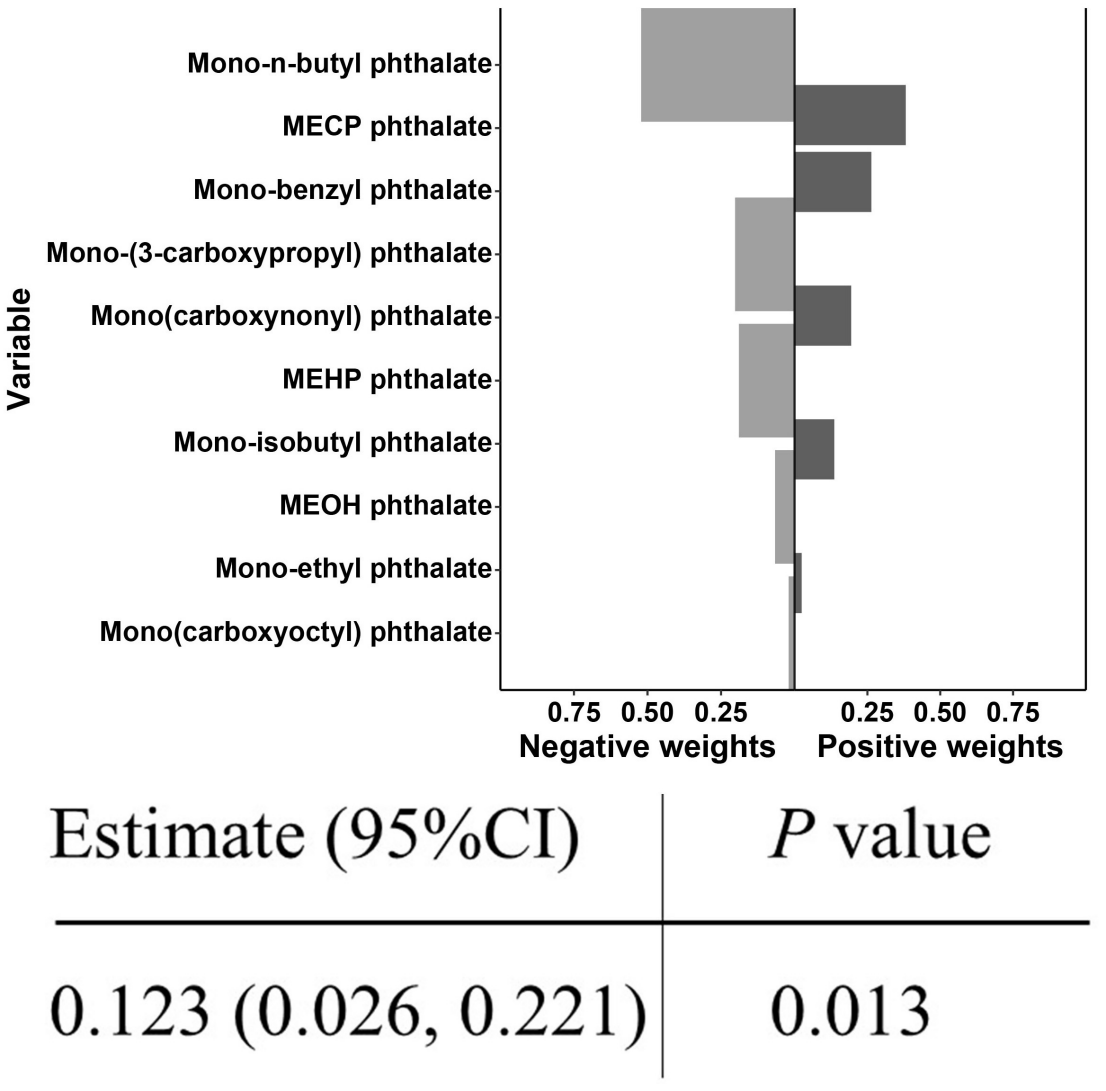
Joint effect of PAEs mixture on CircS and weight of each phthalate according to quantile g-computation.

In the quantile g-computation model, the combined exposure to PAEs was significantly positively correlated with CircS (Estimate = 0.123, *P* < 0.05). The weights of individual PAEs are illustrated in Figure 5, where darker bars on the right side (positive) indicate that the overall effect is positive, with the highest positive weight attributed to MECP phthalate, followed by Mono-benzyl phthalate and Mono(carboxynonyl) phthalate.

## Discussion

Previously, there were limited reports on the effects of PAEs on CircS in epidemiological research. This study analyzed the association between the exposure levels of ten PAEs and CircS within the population, revealing a linear positive correlation between MECP phthalate, MEHP phthalate, and Mono-benzyl phthalate and CircS.

The harmful effects of PAEs on circadian rhythms remain largely unknown. Evidence from animal experiments has preliminarily confirmed the association between PAEs and circadian rhythms. In Caenorhabditis elegans experiment, exposure to phthalates was found to decrease or even completely disrupt the amplitude of circadian rhythms (13). In fruit fly studies, exposure to phthalates perturbed the expression of genes associated with circadian rhythm control and modified the circadian rhythms of adult fruit flies (11). Additionally, exposure to phthalate homologs has been observed to induce changes in circadian rhythms in zebrafish (19). Phthalates can inhibit the expression of the core circadian transcription factor BMAL1 (11), Suppression of BMAL1 expression can lead to a range of physiological abnormalities, including the cancellation of circadian rhythmic behavior, disturbances in the sleep-wake cycle, disruptions in sleep homeostasis, and depressive-like phenotypes (20, 21). In a recent epidemiological study, an association was identified between phthalate exposure and elevated glucose in the components of metabolic syndrome among adult males (22). Similarly, we also uncovered substantial connections between PAEs and the components of CircS. For instance, exposure to high concentrations of MECP phthalate substantially increased the risk of central obesity, elevated glucose, low HDL-C, and depression, aligning with multiple cross-sectional studies from the U.S. (23–25). Prospective cohort studies have also identified an association between PAEs and components of circadian rhythms. In the Women's Health Across the Nation Multipollutant Study, certain phthalate metabolites were identified as being linked to a higher occurrence of diabetes over a 1-year period (26). Moreover, prospective cohort study conducted at five locations in the U.S. have shown a connection between phthalate exposure and an elevated risk of postpartum depression (27), with another U.S. prospective study confirming that phthalate exposure can contribute to postpartum depression (28). The above research findings provide strong scientific evidence for the link between PAEs and CircS.

MEHP is a breakdown product of DEHP in the human gastrointestinal tract, and both DEHP and MEHP phthalate are frequently detected in human blood, urine, and breast milk, where they can induce toxic effects on the endocrine system and contribute to a variety of metabolic disorders (29). Epidemiological research has demonstrated that exposure to higher levels of DEHP is associated with an increased likelihood of moderate to severe sleep disturbances (30) and a higher risk of sleeping < 7, 6, or 5 h (31). Furthermore, animal studies have demonstrated that DEHP exposure can regulate the transcription levels of circadian rhythm genes in mice (14, 32), and lead to reduced expression of Bmal1 in the liver, thereby affecting circadian rhythms (33). Compared to DEHP, MEHP phthalate can enter the systemic circulation more readily, facilitating its distribution throughout the body and potential bioaccumulation, which may result in greater toxicity than DEHP (34). Therefore, it is reasonable to hypothesize that MEHP phthalate may increase the risk of CircS. Regrettably, definitive experimental evidence regarding the effects of MECP phthalate and Mono-benzyl phthalate on CircS has not yet been reported.

BKMR and quantile g-computation can all be utilized to analyze the health effects of chemically related substances. BKMR is particularly flexible in modeling joint effects, allowing for potential interactions and nonlinear effects among mixture components; however, it does not provide estimates of the impact of mixed exposure on outcomes (35). The quantile g-computation effectively infers the overall impact of mixtures and individual contributions without needing to assume a priori whether the effects are positive or negative (36). Therefore, employing BKMR and quantile g-computation allows for consideration of their respective advantages and limitations, clarifying the interactions among chemical mixtures. In this study, significant positive correlations between PAE mixtures and CircS were observed across two exposure models, suggesting that PAE mixtures exposure may increase the risk of CircS. Therefore, this study recommends minimizing the use of personal care products containing phthalates, such as certain cosmetics and fragrances, as well as avoiding the use of plastic containers for food packaging (37). Additionally, it advocates for replacing fast food, processed snacks, and canned foods with fresh and healthy alternatives (37, 38) to lower phthalate exposure in the target population.

Despite the advantages of this study in exploring the relationship between PAEs exposure and circadian rhythm syndrome, several notable limitations must be acknowledged. Firstly, the cross-sectional nature of this study prevents the determination of a causal link between PAEs exposure and CircS. Although the discussion section offers speculative biological mechanisms, cohort studies and animal experiments are crucial for further validating our findings. Second, although urinary analysis is considered an appropriate method for assessing phthalate exposure (15, 39, 40), the relatively short biological half-life of phthalates, combined with the fact that NHANES evaluates phthalate metabolites based solely on a single measurement and lacks information on exposure duration, may lead to an unreliable reflection of habitual contact levels in urinary diester metabolite concentrations (26). Consequently, the potential for exposure misclassification cannot be entirely ruled out. Finally, potential confounding factors, such as exposure to other endocrine-disrupting chemicals, may influence the validity of the study results.

## Conclusion

Both individual exposure to MECP phthalate, MEHP phthalate, and Mono-benzyl phthalate, as well as mixed exposure to PAEs, may increase the risk of CircS. Further research is necessary to validate our findings and to elucidate the potential pathophysiological mechanisms by which various phthalate metabolites contribute to CircS.

## Data Availability

Publicly available datasets were analyzed in this study. This data can be found here: https://www.cdc.gov/nchs/nhanes/index.html.
