# Supplementary material for: Associations of urinary phthalate metabolites with Circadian Syndrome: evidence from NHANES
Source: Front Public Health. 2025 Jun 27;13:1597489. doi: 10.3389/fpubh.2025.1597489 (PMC12245799; doi:10.3389/fpubh.2025.1597489)
Supplement: Supplementary file 1 [file Data_Sheet_1.docx]

Supplementary Material

# Supplementary Figures and Tables

## Supplementary Table

**Table S1** Distribution of PAEs (N = 2519), NHANES, USA, 2013–2018.

| Characteristic (ng/mL) | Total  (n = 2519) | CircS  (n = 1137) | Non-CircS  (n = 1382) | *P* value |
| --- | --- | --- | --- | --- |
| Mono(carboxynonyl) phthalate | 1.80 (0.90-3.50) | 1.90 (1.00-3.60) | 1.80 (0.90-3.40) | 0.355 |
| Mono(carboxyoctyl) phthalate | 7.60  (3.60-21.70) | 7.80  (3.90-22.70) | 7.50  (3.50-21.40) | 0.390 |
| MECP phthalate | 8.60  (4.50-15.30) | 9.00  (5.20-15.80) | 8.30  (4.10-15.10) | 0.047 |
| Mono-n-butyl phthalate | 9.70  (4.90-17.70) | 10.50  (5.10-18.70) | 9.30  (4.70-17.30) | 0.099 |
| Mono-(3-carboxypropyl) phthalate | 1.20 (0.60-2.60) | 1.30 (0.60-2.70) | 1.20 (0.60-2.60) | 0.479 |
| Mono-ethyl phthalate | 29.90  (12.80-81.50) | 29.60  (13.40-89.40) | 30.40  (12.60-75.0) | 0.536 |
| MEHP phthalate | 5.20 (2.60-9.80) | 5.40 (2.90-9.80) | 5.00 (2.40-9.80) | 0.101 |
| Mono-isobutyl phthalate | 8.00  (3.70-14.40) | 7.90  (3.60-13.40) | 8.00  (3.70-14.90) | 0.402 |
| MEOH phthalate | 3.50 (1.70-6.40) | 3.60 (1.90-6.40) | 3.50 (1.60-6.30) | 0.154 |
| Mono-benzyl phthalate | 3.70 (1.60-8.50) | 3.80 (1.70-8.80) | 3.60 (1.60-8.40) | 0.238 |

## Supplementary Figure

##
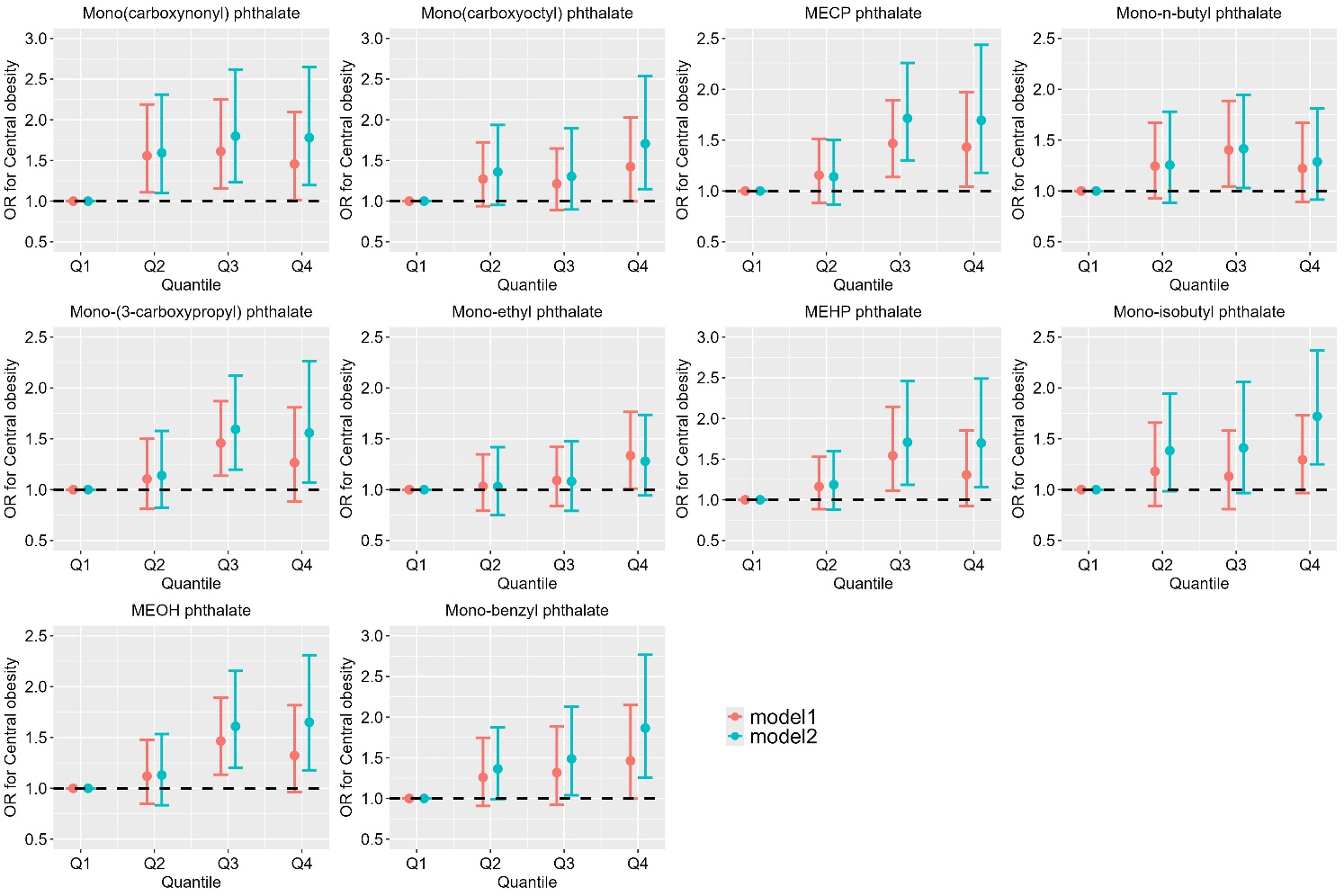


Supplementary Figure 1. Association between single urinary phthalate metabolite concentration and central obesity (N = 2519). NHANES, USA, 2013–2018. Model 1: The unadjusted model. Model 2: Adjusted for age, gender, educational attainment, race, alcohol consumption, physical activity, and cotinine levels.

##
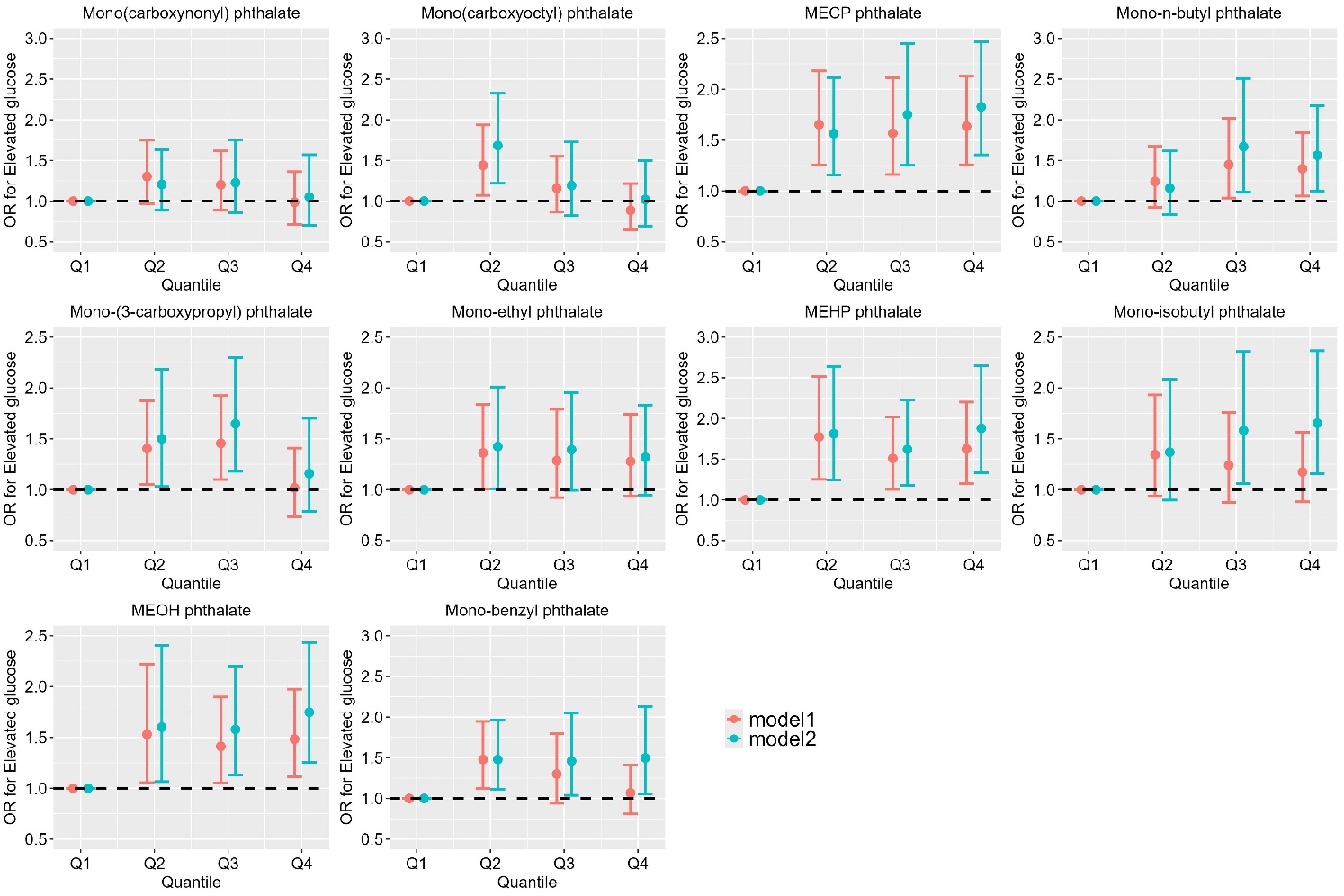


Supplementary Figure 2. Association between single urinary phthalate metabolite concentration and elevated glucose (N = 2519). NHANES, USA, 2013–2018. Model 1: The unadjusted model. Model 2: Adjusted for age, gender, educational attainment, race, alcohol consumption, physical activity, and cotinine levels.

##
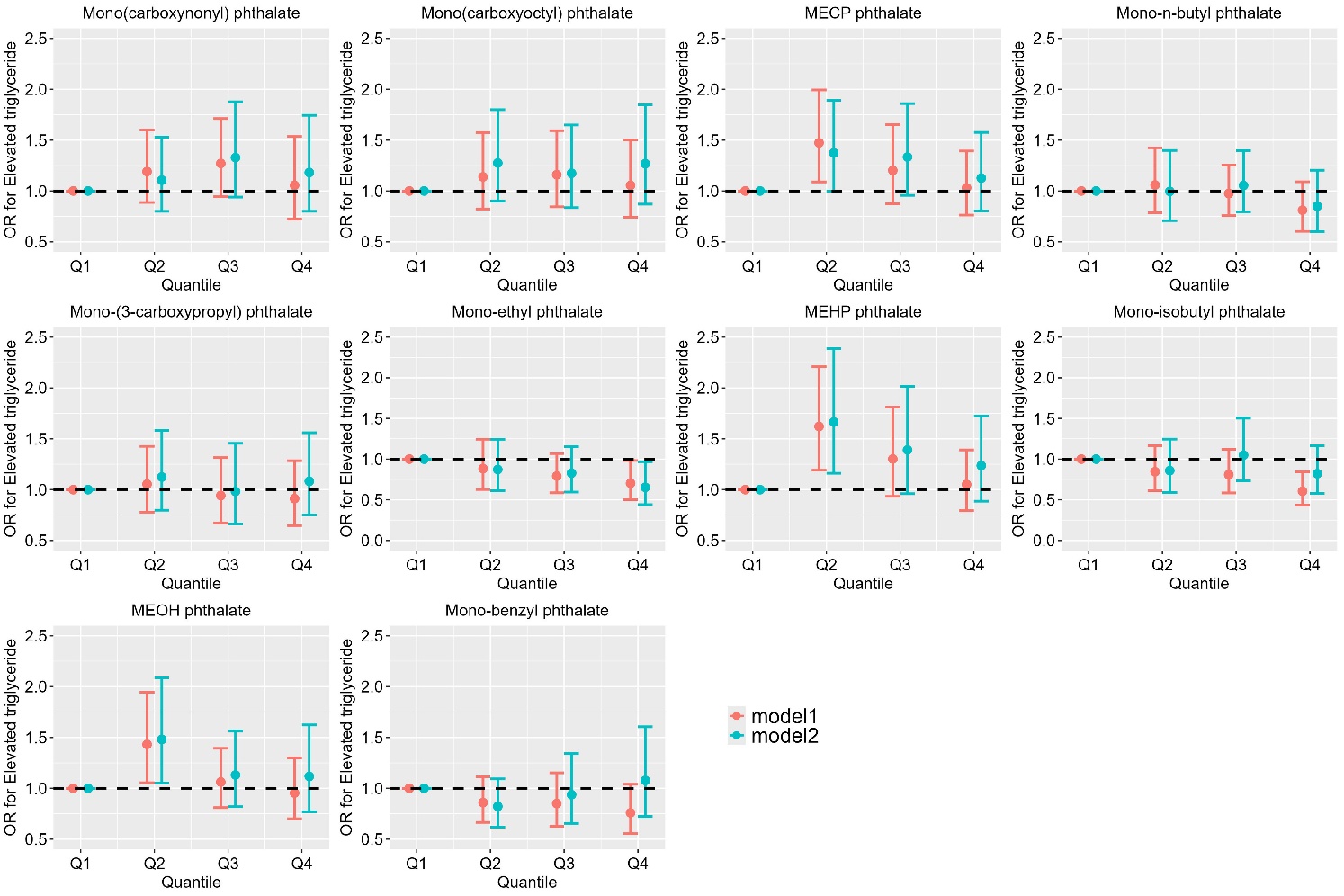


Supplementary Figure 3. Association between single urinary phthalate metabolite concentration and elevated triglyceride (N = 2519). NHANES, USA, 2013–2018. Model 1: The unadjusted model. Model 2: Adjusted for age, gender, educational attainment, race, alcohol consumption, physical activity, and cotinine levels.

##
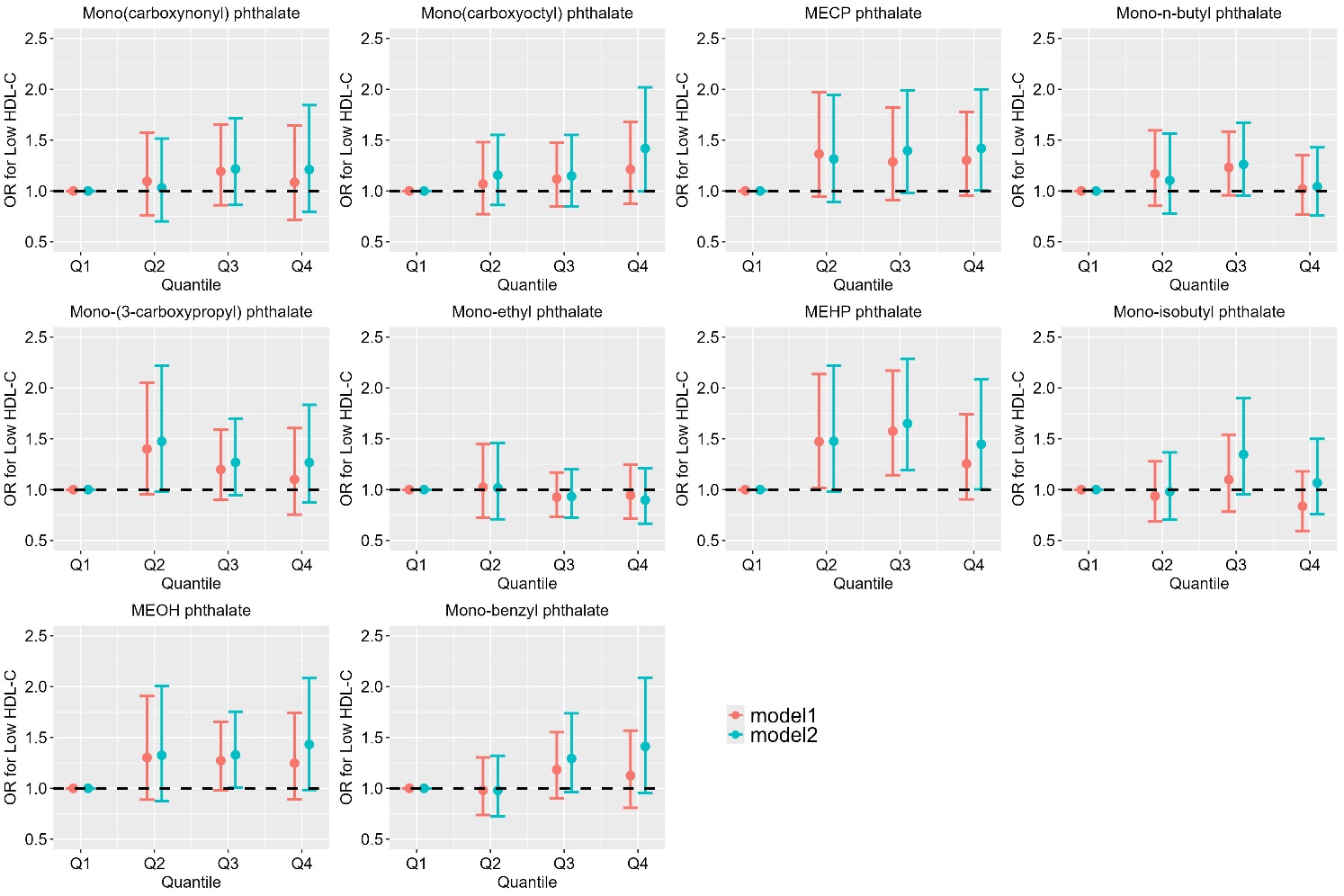


Supplementary Figure 4. Association between single urinary phthalate metabolite concentration and low HDL-C (N = 2519). NHANES, USA, 2013–2018. Model 1: The unadjusted model. Model 2: Adjusted for age, gender, educational attainment, race, alcohol consumption, physical activity, and cotinine levels.

##
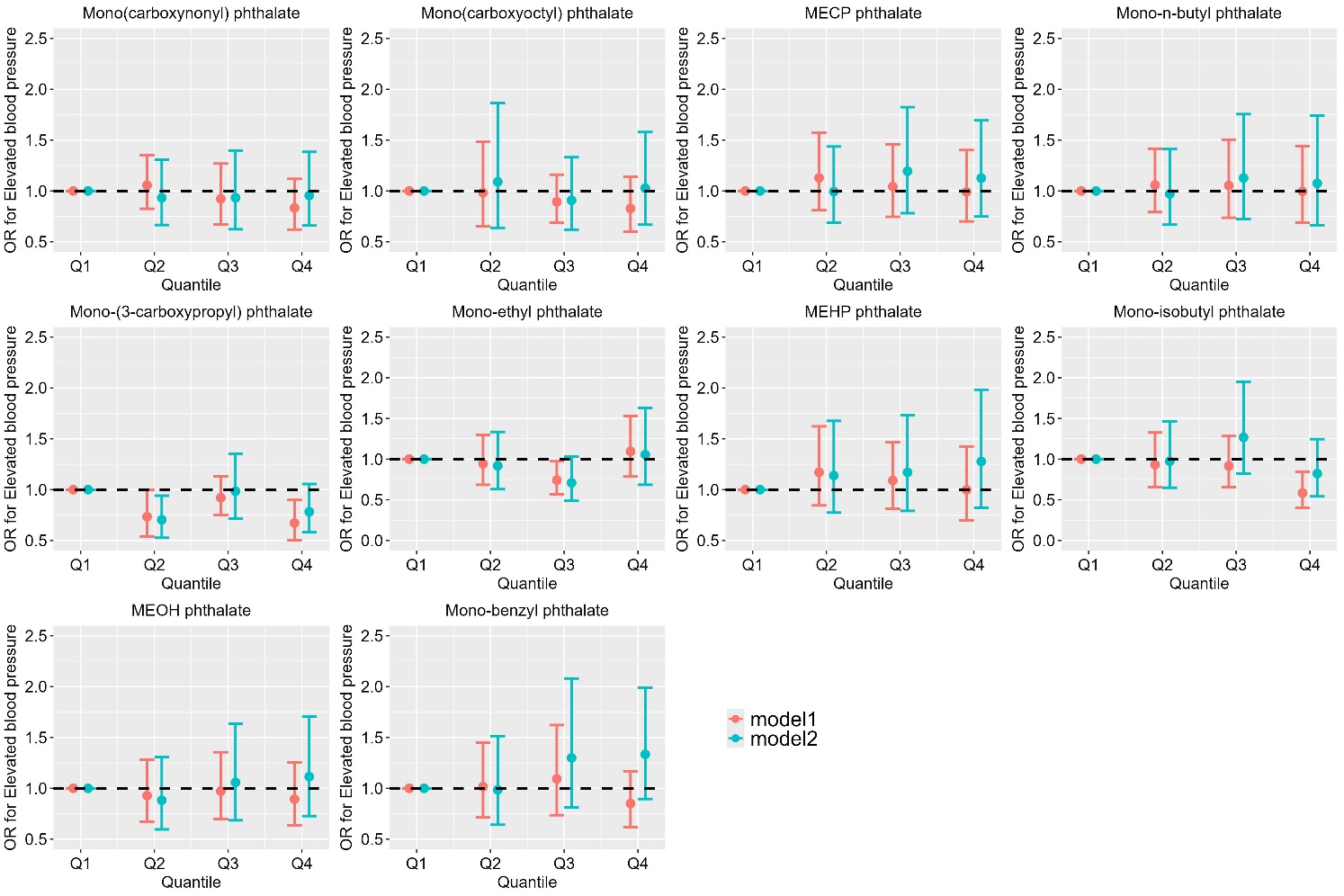


Supplementary Figure 5. Association between single urinary phthalate metabolite concentration and elevated blood pressure (N = 2519). NHANES, USA, 2013–2018. Model 1: The unadjusted model. Model 2: Adjusted for age, gender, educational attainment, race, alcohol consumption, physical activity, and cotinine levels.

##
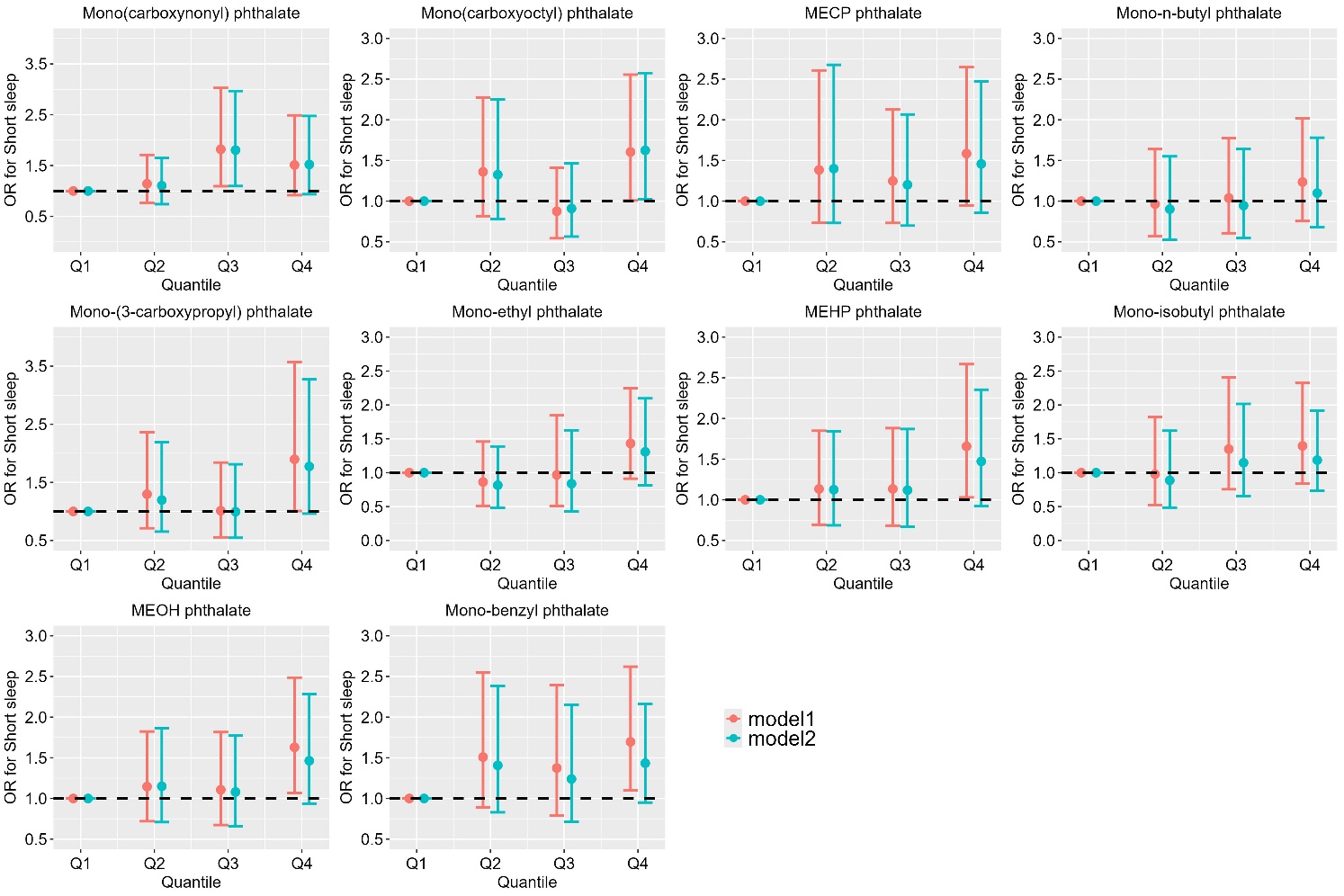


Supplementary Figure 6. Association between single urinary phthalate metabolite concentration and short sleep (N = 2519). NHANES, USA, 2013–2018. Model 1: The unadjusted model. Model 2: Adjusted for age, gender, educational attainment, race, alcohol consumption, physical activity, and cotinine levels.

##
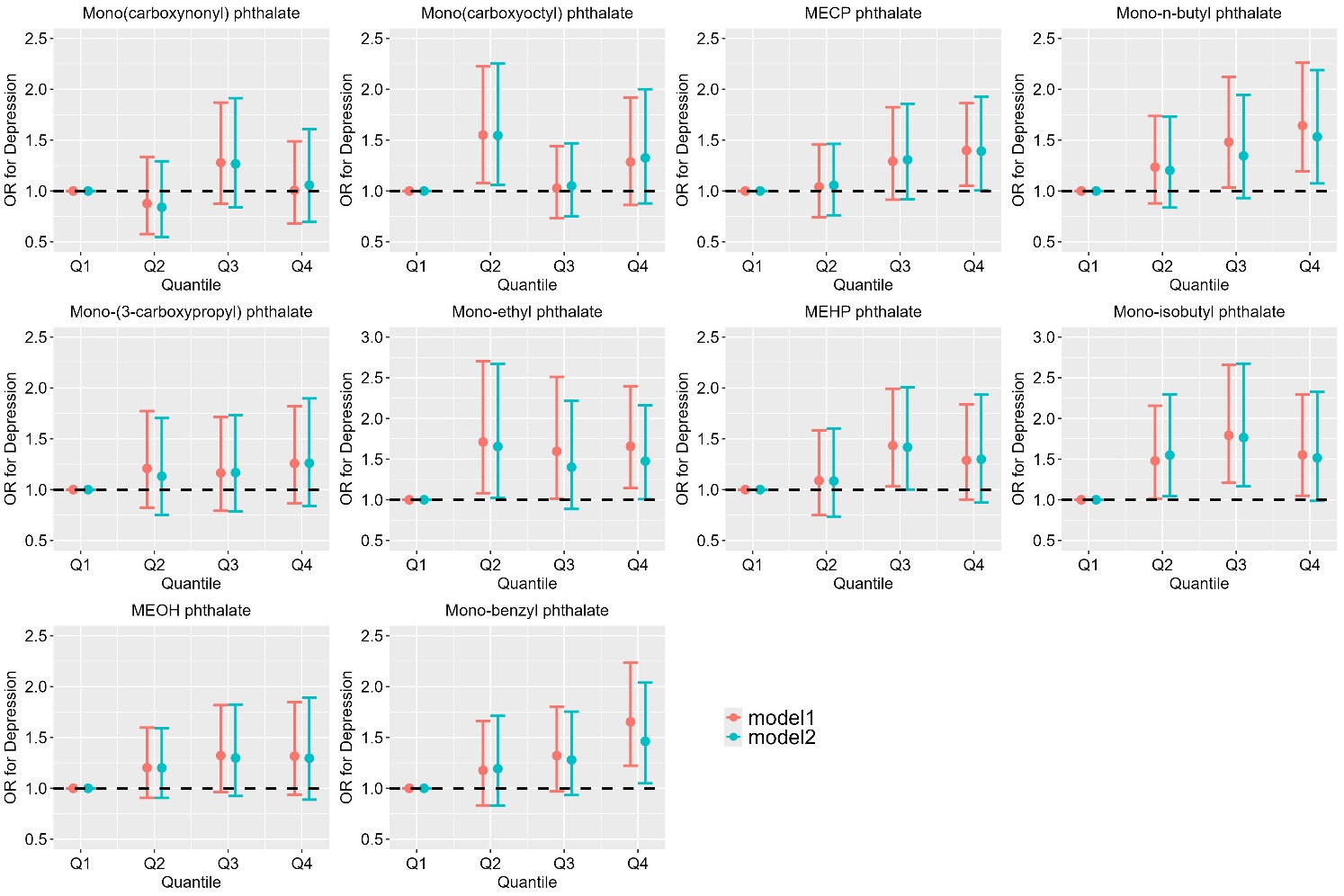


Supplementary Figure 7. Association between single urinary phthalate metabolite concentration and depression (N = 2519). NHANES, USA, 2013–2018. Model 1: The unadjusted model. Model 2: Adjusted for age, gender, educational attainment, race, alcohol consumption, physical activity, and cotinine levels.


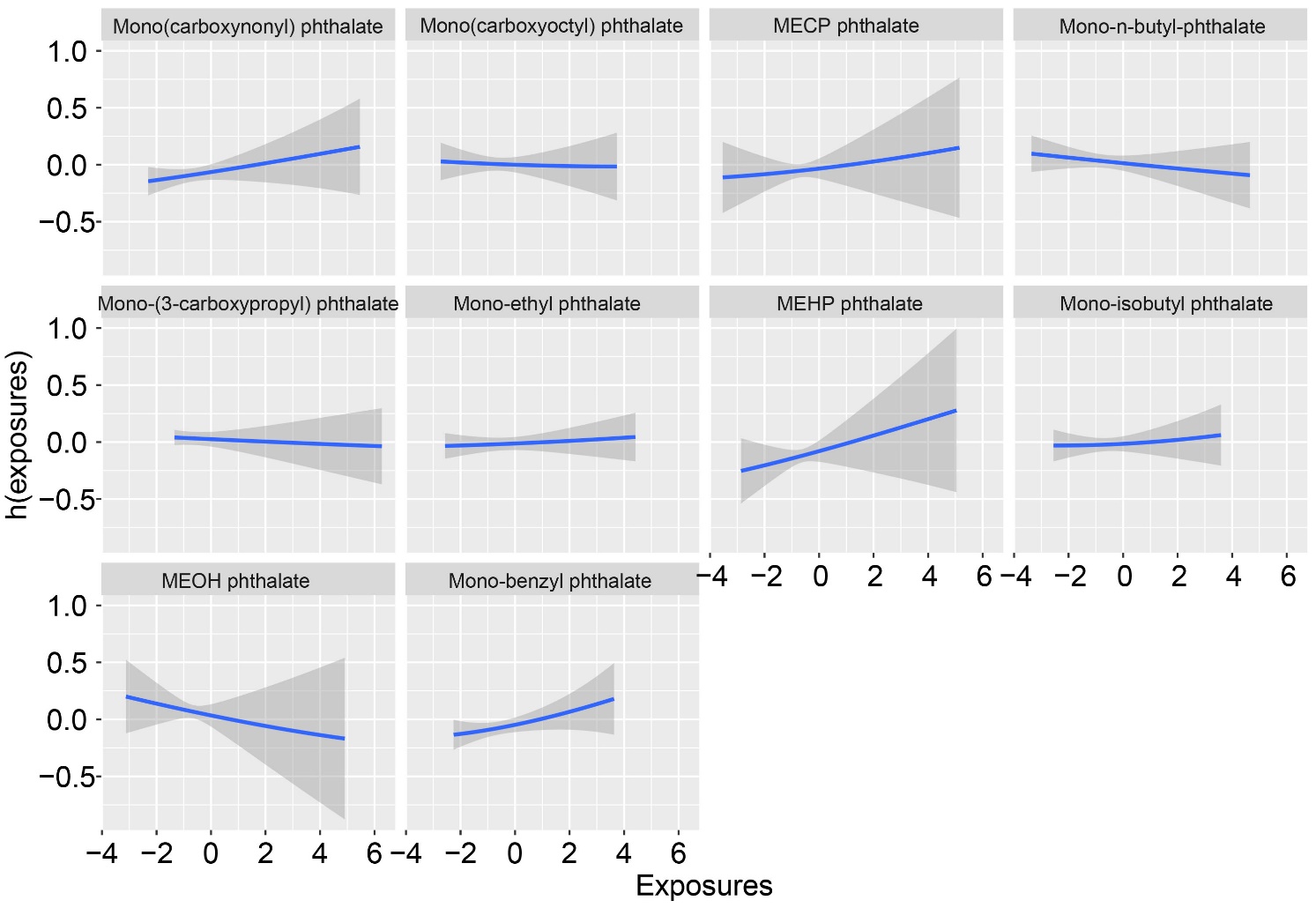


**Supplementary Figure 8.** The dose-response relationship of single phthalate with CircS, when other phthalates were fixed at the 25th percentile. Model was adjusted for age, gender, educational attainment, race, alcohol consumption, physical activity, and cotinine levels.
